# Medical History from the Earliest Times

**Published:** 1892-07-30

**Authors:** E. T. Withington


### Medical History from the Earliest Times.

By E. T. Withington, M.A., M.B. Oxon.

### XIX.—CELSUS AND ANCIENT SURGERY.

Aulus Cornelius Celsus, who has somewhat extravagantly been called the Cicero of physicians and the Latin Hippocrates, is the most important of Roman medical writers. But he does not seem to have been a member of the profession, and probably intended his treatises on agriculture, medicine, war, and rhetoric, to form a compendium of all the knowledge requisite for a wealthy citizen, who had a farm and slaves to superintend, and might hold a public office. He may himself have been in such a position, and may have acquired some practical knowledge in the "Valetudinaria" or slave infirmaries which were maintained by rich landowners, but, except that he lived in the Augustan age, we practically know nothing of him. The historical importance of his work, "De Medicina," consists in the excellent but too brief sketch of medical history with which it commences, in its notices of more than seventy Greek physicians whose works have perished, and in the last two books which give us some idea of the progress of surgery since the time of Hippocrates. It may, therefore, be conveniently taken as the text for a brief account of some of the more important aspects of ancient surgery, a subject which may be further simplified by connecting it with the names of the three greatest of Greek surgeons, Heliodorus, Archigenes, and Antyllus.

Celsus tells us that medicine was divided at at Alexandria into three branches, one curing by diet, another by drugs, and a third by hand, which were called by the Greeks dietetic, pharmaceutic, and chirurgic respectively. This was, no doubt, mainly a theoretical division such as Celsus follows in his own work, but he repeats elsewhere that surgery began to have its special professors at Alexandria, and we have already noticed traces of such a division in yet earlier times. In the Roman period the physician and surgeon, the *medicus clinicus* and *chirurgicus*, were clearly distinguished, and Galen, writing on operative medicine, says he would probably have known more of the subject had he stayed in Asia, but on coming to Rome he found that such matters were left to "those called surgeons," and he had followed the general example.

The golden age of Greek surgery was the close of the first century, and it is marked by the names of Heliodorus and Archigenes, both of whom are mentioned by Juvenal; Antyllus probably flourished at least a century later, for, though largely quoted by Oribasius, he is not noticed by Galen.

Heliodorus seems to have been a pure surgeon, for all the surviving fragments of his writings deal with that art. He was specially famous for his knowledge and treatment of injuries of the head, and for the operation for hernia, and was, perhaps, the first to treat stricture by internal urethrotomy; but his name may most conveniently be connected with the history of the methods used by the ancients for checking hæmorrhage. The Hippocratic writers knew nothing of ligature, and treated hæmorrhage by cold, pressure, styptics, and sometimes by the actual cautery; but so obvious a method of closing a bleeding vessel could not long remain untried, and it was probably introduced by the Alexandrine anatomists. Celsus recommends that an injured vessel should be tied in two places, and divided between them, but it is Heliodorus who gives us the first account, not only of terminal ligature, but also of the supposed modern invention of torsion. Speaking of the operation for hernia, he says: "We ligature the larger vessels, but as for the smaller ones we catch them with hooks, and twist them many times, thus closing their mouths." Galen at a later period even tells us that the best shop at which to buy ligatures was in the Via Sacra, between the temple of Rome and the Forum.

Archigenes was equally great as physician and surgeon. He described the varieties of the pulse with even more minuteness than Galen, and suggested that the different kinds of pain might serve to indicate the organs affected; he first drew the important distinction between primary and secondary symptoms in disease, and made the earliest attempt to classify mineral waters according to their composition; but he is especially memorable in connection with the history of amputation. The early Greeks shrunk from that operation, partly, no doubt, because of the formidable hæmorrhage, but partly also from the horror with which they, like the Arabs, looked upon any form of mutilation, the effects of which might, they thought, be continued in a future life. Hippocrates does not mention amputation in its true sense, but in cases of gangrene, when a line of demarcation has been formed, and the irretrievable loss of the limb is evident to the patient as well as to the surgeon, the latter may assist nature by removing the dead part, carefully avoiding to cut into living tissue, for this may cause fatal syncope. Some slight advance was made by the Alexandrines, but Celsus still describes amputation as "the last sad remedy," lawful only in cases of gangrene, though the circular incision is now made rather through living than dead tissue, and the soft parts are drawn back as far as possible before dividing the bone. But in the hands of Archigenes the operation assumes quite a modern shape. The indications include not only gangrene, but chronic ulcers, malignant tumours, severe injury, and great deformity. In some cases the whole part to be removed should be sprinkled with cold water and bandaged, the limb being then tightly constricted with a cord above the point of amputation; where this is not practicable, the chief arteries going to the part should be cut down upon and tied. Rubber bandages were then unknown, or Archigenes might have anticipated the "bloodless" method of Professor Von Esmarch.

Antyllus, like Archigenes, seems to have been a general practitioner, for, besides purely surgical subjects, he wrote on hygiene, the choice of proper sites for houses, and the action of purgatives and other drugs. His name is probably better known than that of either of his predecessors, owing to its connection with the earliest operation for aneurism; this, however, is described in most works on surgery, and our brief remaining space will be better occupied by a historical sketch of the treatment of cataract. When the lens of the eye is opaque, it may be removed from the axis of vision in at least four ways, all of which seem to have been known to the Greeks. (1) It may be simply depressed or "couched." This operation, though now abandoned, is of great antiquity, having been known to the Egyptians and Hindoos, and was probably the only one practised up to the Christian era. (2) It may be extracted entire, a method first mentioned by Galen, apparently as a recent invention, for he says: "Some have taken in hand to remove cataract also." (3) The lens may be broken up and left to be absorbed. Celsus notices the division of cataract, though only as a preliminary to couching, but Galen clearly describes the operation, which he rightly confines to soft cataracts, or, as he terms it, those of more serous humor. (4) The lens may be broken up and at once removed by suction. This operation, recently introduced into modern medicine, was long practised in Persia, and, according to Albucasis, was invented there in his time (eleventh century); but Rhazes, who was himself a Persian, and who had special inducements to study the treatment of cataract, attributes the earliest mention both of extraction and suction to Antyllus, remarking: "Antyllus said 'Some also have made an opening under the pupil, and have extracted the cataract; this can be done when the cataract is small, but if large it cannot be extracted, for the humor comes out with it. And some have used a glass instrument (concilum vitreum), and by sucking it have sucked out the opaque matter (albugineum).'"

